# A prospective multi-center cohort study of acute non-displaced fractures of the scaphoid: operative versus non-operative treatment [NCT00205985]

**DOI:** 10.1186/1471-2474-7-41

**Published:** 2006-05-11

**Authors:** Boris M Pfeiffer, Matthias Nübling, Hartmut R Siebert, Michael Schädel-Höpfner

**Affiliations:** 1 AO Clinical Investigation and Documentation, Clavadelerstrasse 8, CH-7270 Davos Platz, Switzerland; 2 FFAS, Freiburg research centre for occupational and social medicine, Bertoldstrasse 27, D-79098 Freiburg, Germany; 3 Department of Hand-, Plastic- and Trauma Surgery, Diakonie-Krankenhaus, Postfach 10 06 54, D-74523 Schwäbisch Hall, Germany; 4 Department of Trauma and Hand Surgery, University Hospital, Moorenstrasse 5, D-40225 Düsseldorf, Germany

## Abstract

**Background:**

Acute scaphoid fractures are common in active adults and do lead to reasonable time lost to work. One important goal of treatment is early return to work or sport. On this background, the adequate treatment of non-displaced acute scaphoid fractures is still under discussion. The aim of this study is to compare time to return to previous activity level comparing surgical versus non-surgical treatment of non-displaced acute scaphoid fractures.

**Methods/Design:**

The study is designed as a non-randomized multiple center cohort study including 12 sites in Germany and Austria. The inclusion period is planned to be 12 months with a follow up of 6 months. Allocation to operative or non-operative treatment is choosen by the patient together with his treating surgeon. The primary outcome is time to return to previous activity level adapted for loading of the wrist in daily life as measured by a newly developed questionnaire (PLDL-wrist). Factors identified a priori to be associated with the outcome, e.g., poverty status, age, education, smoking status, gender, and occupation, are measured to ensure adequate control for their potential confounding effects.

**Discussion:**

The rationale and the design of a multiple center cohort study are presented. As it is not considered feasible to randomize patients in this study, potential confounding effects need to be controlled adequately.

## Background

Acute scaphoid fractures mainly occur in active adults, many of whom work as laborers or are involved in competitive athletics; therefore, returning to work or sport is an important goal of treatment. They are the most common fractures of the carpal bones. Time to union of acute scaphoid fractures ranges from 6 to 18 weeks depending on fracture type, localization of the fracture, degree of displacement, and additional soft tissue injury [1-4].

Cast immobilization remains the standard treatment despite the fact that about 20% of fractures fail to heal with conservative treatment[1,5,6]. It is well documented that the rate of nonunion of acute scaphoid fractures is higher for proximal and dislocated fracture types [7-10]. In displaced scaphoid fractures, the nonunion rate rises up to 46%. Advances in the technique and in implants developed for the operative fixation of acute scaphoid fractures have made it technically easier with a higher rate of union [5,11-16]. Operative treatment is recommended for all displaced, unstable and complex fracture types [2,5,13-15,17-20]. In non-displaced acute scaphoid fractures treated by limited access and screw fixation the union rate has been reported as high as 100% [4,5,11-14,21].

The adequate treatment of non-displaced acute scaphoid fractures is still under discussion. A few prospective studies with a limited number of patients and imprecise definitions of fracture type and union were published in 2000 and 2001 [11,12,16]. Despite favoring screw fixation, two recent randomized controlled trials (RCTs) that used 'return to work' (RTW) as an outcome [12,22] did not provide a clear definition or did not define who determined return to work. Neither study administered a patient-derived quality of life measure specific to the wrist like the Disability of the Arm, Shoulder and Hand (DASH) questionnaire [23-25]. In the Cochrane library, there are no systematic reviews or meta-analyses on the treatment of acute scaphoid fractures. Considering the problems associated with prolonged cast immobilization, the complications of non-operative fracture treatment and social/economic factors, it seems appropriate to evaluate the possible advantages of routine operative treatment with a less invasive screw fixation technique for non-displaced acute scaphoid fractures.

**Figure 1:**
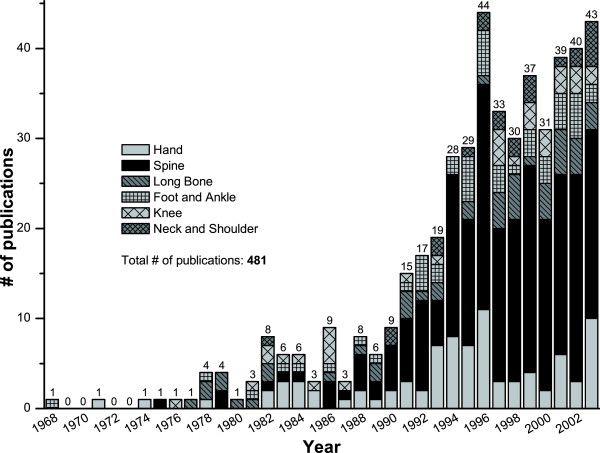
**Number of publications of studies with RTW as outcome parameter**. Pubmed-search from 1966 – 12/2003 using the search strategies: (1) [('return' OR 'day* lost' OR 'disability') AND 'work'] OR 'sick leave'; Limit: 'Clinical Trial' (2) {[('return' OR 'day* lost' OR 'disability') AND 'work'] OR 'sick leave'} AND 'Orthopaedic Procedures [mh]'. The abstracts of the retrieved publications were scanned to identify clinical studies using either 'return to work' or 'time of sick leave' as primary or secondary outcome. The found studies were categorised into one of the following 6 groups: 'Hand', 'Spine', 'Long Bone', 'Foot and Ankle', 'Knee' or 'Neck and Shoulder'.

The determination of RTW is of growing importance as an outcome in clinical studies in the field of trauma and orthopedic surgery and its application has increased in recent years (Figure 1). One limitation of this outcome is, however, that return to work is applicable for employees only what implies, that RTW cannot be measured in e.g., students, housewifes and -men, unemployed or retired. Also, a wide range of definitions of RTW can be found throughout the literature. The different concepts of RTW have been devided into three groups [26]: (1) Cumulatively, counting all days lost from work beginning with the date of injury, (2) categorically, e.g., RTW ever (yes/no); working at time × (yes/no) or (3) continuously, as time-to-RTW. These concepts have in common, that no information is given nor is any adjustment made for, whether the physical load after RTW differs from the load before the event observed. Therefore, no conclusions can be drawn, whether any functional limitations after RTW are present. To overcome the described limitations in the context of this study, an additional instrument was needed to adapt the outcome RTW and expand its meaning to determine the physical load in daily life, including work and household duties as well as leisure time.

We implemented a prospective cohort study to compare patients with non-displaced scaphoid fractures undergoing operative treatment with those who have non-operative care, addressing several questions: Is the time to return to previous activity level different between those undergoing surgery and those treated non-operatively? In addition, it will be determined whether any differences in treatment outcomes, including functional status as derived from the DASH and SF-36 [27-31], complication rate, range of motion, grip strength, and patient evaluation of pain and overall satisfaction can be seen between the patient groups. Such knowledge could support surgeons and patients in finding an adequate treatment decision.

Finally, cost-effectiveness of surgical versus non-surgical treatment will be evaluated, focusing on direct, as well as indirect costs of treatment and social/economic factors.

## Methods/Design

**Figure 2:**
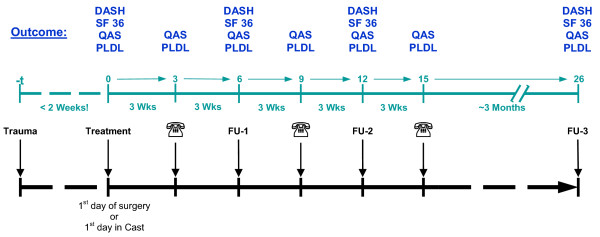
**Follow up procedures**. At 6-weeks, 3-months and 6-months, the determination of the outcomes is done during in-house visits of the patients in the clinic. At 3-weeks, 9-weeks and 15-weeks, the QAS and PLDL-wrist questionnaires are retrieved via telephone interview.

We designed a multi-center prospective cohort study of patients with complete, non-displaced scaphoid fractures receiving either operative or non-operative treatment to compare the time to return to daily activities. The inclusion period will be approximately 12 months. Follow-up examinations (Figure 2) are planned at 6-weeks, 3-months and 6-months following onset of treatment. Additionally, telephone interviews are planned at 3-, 9- and 15 weeks.

The clinical investigational plan was approved by the ethics committee of the Medical Faculty of Philipps-University Marburg.

### Study design

The study is designed as a non-randomized cohort study, involving multiple sites with multiple surgeons and potentially differing patient populations. The chosen design implies a potential for bias and uncontrolled confounding. This will be addressed in the method of data collection and data analysis (see below). The eligibility criteria do only allow patients to be included, that do have isolated, complete fractures of the mid third of the scaphoid without signs of displacement or comminution visible in CT scan, while patients with any additional fracture of other sites are excluded, leading to a very homogenous injury pattern an thus minimizing residual "confounding by indication".

### Patients

Adult patients with isolated, acute (no more than 2 weeks between injury and treatment) complete fracture of the mid third of the scaphoid, without any signs of displacement or comminution visible in CT scan, are eligible for this study. Patients meeting the inclusion and exclusion criteria (Table 1) are identified by the participating centers and will be informed about the study. Patients are recruited from 10 clinics in Germany and 2 clinics in Austria (Table 2). All patients need to sign an informed consent form before entering the study.

### Treatment allocation

The decision on whether to choose an operative or non-operative treatment procedure is taken by the patient together with his treating surgeon. The participation in the study is completely independent of this decision and does not influence the choice of treatment procedure. Patients are classified as operative or non-operative according to the initial treatment decision taken during the first two weeks following injury.

### Interventions

Operative treatment (Group A) will be performed using minimally invasive palmar or dorsal access. Retrograde as well as antegrade screw insertion is up to the choice of the performing surgeon. The fracture fixation is done in free hand technique, using a cannulated screw. Postoperative splint immobilization should not exceed 1 week.

Non-operative treatment (Group B) will be performed with short arm plaster cast including first carpo-metacarpal joint but leaving first metacarpal-phalangeal joint and all fingers free. Immobilization for 6 weeks is recommended, further immobilization if necessary.

Patients in both groups are supposed to receive 10 sessions of physical therapy.

### Outcome assessment

This study focuses on differences in time to return to daily activities and patient derived quality of life instruments between the two groups. These outcome parameters will be assessed by means of questionnaires.

### Primary outcome measures

(1) Questionnaire on physical loading of the wrist in daily life (PLDL-wrist). The PLDL-wrist (Additional file 1: German version, Additional file 2: English translation) is a newly developed 17-item questionnaire, based on the German translation of the DMQ [32]. Each of the items can be answered by either 'YES' or 'NO', leading to a maximum score of 17. At baseline all patients will be interviewed to get the baseline PLDL-score of the last week before the injury. t_0_, being defined as the day of onset of treatment, will be given the baseline PLDL-score. The questionnaire will be repeated at several follow-ups according to the schedule in Figure 2. The individual score at every interview will be expressed as a percentage of the baseline score (PLDL_xweeks_/PLDL_Baseline _* 100) and plotted over time. The endpoint is defined as 'Time to reach the individual 80%-threshold'. To determine the time point at which the 80%-threshold is reached, the change between the first score value exceeding the 80%-threshold and the prior score value is linearized. The intercept of this line and the 80%-threshold represents the linear estimation of the first day of return to previous activity level (t_return_).

(2) Questionnaire on the activity status (QAS). The QAS differentiates between the categories (A) return to work [full; partly; no; not applicable], (B) return to household duties [full, partly, no] and (C) return to leisure activities [full, partly, no]. Using the QAS will allow us to include patients who are not formally employed (e.g. students, housewives and -men, self- or unemployed). At baseline all patients will be asked for their activity status in the last week before the injury. This will be repeated according to the schedule in Figure 2. A patient has returned to previous activity level either when he or she has returned to full work **and** returned to full household duties or – for those not being employed – has returned to full household duties only. Rates of return to previous activity level will be calculated for each of the follow-up periods. Leisure activities are not included in this definition; these are evaluated as secondary outcomes, however.

### Secondary outcome measures

(a) Disability of the Arm, Shoulder and Hand (DASH). Functional status will be measured by the DASH questionnaire at every follow up (Figure 2.)

(b) Short Form 36 (SF-36). Quality of Life will be determined, using the SF-36 questionnaire according to the schedule in Figure 2.

(c) Patient estimation of pain and patient satisfaction. Overall satisfaction with the current condition will be determined by the patient, using a visual analog scale (VAS). Patients are asked to set a vertical mark on the VAS for question 1: How would you estimate the pain in the injured hand, ranging from 'No Pain' to 'Worst possible Pain'?; and question 2: How would you estimate your satisfaction with your current condition ranging from 'very satisfying' to 'absolutely unacceptable'?

(d) Patient overall satisfaction with treatment will be determined by the patient, using a 6-step scale ranging from 'very good' to 'non-satisfactory'.

(e) Range of Motion (ROM) of the wrist will be measured with a goniometer at all follow-up examinations by an independent investigator.

(f) Grip strength will be measured with a Jamar dynamometer, comparing the injured to the non-injured wrist and adjusting for dominance.

(g) Radiological healing

(h) Complication rate

### Hypothesis

Those patients suffering an acute complete fracture of the mid third of the scaphoid bone without any dislocation or comminution visible on the CT-scan who are treated surgically with cannulated screw fixation will return to their previous activity level sooner than patients treated non-operatively with short arm cast immobilization.

### Null hypothesis

There will be no difference in terms of return to previous activity level between patients suffering an acute complete fracture of the mid third of the scaphoid bone, without any dislocation or comminution visible on the CT-scan who are treated surgically with cannulated screw fixation and patients treated non-operatively with short arm cast immobilization.

### Sample size calculation

Given what was reported in 2 recent RCTs, it is anticipated that we will detect a significant difference in mean time to return to previous activity level comparing the two groups. However, the primary outcome in these RCTs was return to work, defined as 'time on sick leave' [22] and 'return to full military duty' [12], respectively; who determined the outcome was not defined, nor was the level of posttraumatic physical activity compared with the level prior to the trauma. The 'return to previous activity level' is assumed to be equivalent to the definition of RTW found in the 2 RCTs, but more objective.

According to a survey in the clinics being invited to participate, it can be expected that the odds of patients receiving non operative treatment in relation to those receiving surgery is 1:4 (i.e. there will be 1 patient with short arm cast treatment for each group of 4 patients being operated on).

The sample size calculation for the primary outcome (1) is based on a two-sided two-sample t-test, a significance level of 0.05 and a power of 90%. A difference of 4 weeks between the two groups is likely and a standard deviation of 5 weeks is anticipated. As a 25% loss to follow-up is expected, we recommend a minimum of 112 patients in the cannulated screw group and 28 patients in the short arm cast group.

The sample size calculation for the primary outcome (2) is based on the numbers shown in Table 3: If the sample size in group A is 86 and the sample size in group 2 is 30, the log-rank test for equality of survival curves with a 0.050 two-sided significance level will have a power of approximately 92% to detect the difference between the survival curves specified in the side table.

### Data analysis

All data analysis for the primary outcomes will be done on an intention to treat basis. As the baseline variables shown in Table 4 are known a priori to be associated with either of the two primary outcomes, they will be collected to ensure we adequately control for their potential confounding effects. In the secondary analysis, the outcomes will be adjusted for these confounding factors.

## Discussion

When planning the study, a randomized controlled trial was considered but as the participating surgeons, as well as patients, often have preferences for one of the two treatments under investigation, we decided to design this study as a prospective non-randomized cohort study. However, the design implies a potential for bias and uncontrolled confounding, which needs to be addressed in the method of data collection and data analysis.

## Conclusion

The rationale and the design of a cohort study comparing the time to return to daily activities in patients with non-displaced scaphoid fractures receiving operative treatment versus patients receiving non-operative treatment has been presented.

## Abbreviations

CT – Computer tomography

DASH – Disability of the Arm, Shoulder and Hand questionnaire

DMQ – Dutch musculoskeletal questionnaire

PLDL-wrist – Questionnaire on physical loading of the wrist in daily life

QAS – Questionnaire on activity status

RCT – Randomized controlled trial

ROM – Range of motion

RTW – Return to work

SF-36 – Short form 36

VAS – Visual analog scale

## Competing interests

The author(s) declare that they have no competing interests.

## Authors' contributions

BMP, HRS and MSH were responsible for the design of the study. BMP and MN developed the PLDL-wrist questionnaire and adapted the outcome measures. BMP drafted the manuscript. All authors read an approved the final manuscript.

## Pre-publication history

The pre-publication history for this paper can be accessed here:



## Supplementary Material

Additional File 1 PLDL-wrist German version Click here for file

Additional File 2 PLDL-wrist English translation Click here for file

## Figures and Tables

**Table 1:** Patient eligibility criteria

**Inclusion criteria:**
• Patient agreed with and signed the "informed consent" form
• Adult patients (≥ 18 years old) [Mature Skeleton]
• Patients with isolated, acute (no more than 2 weeks between injury and treatment), non-displaced complete fracture of the mid third of the scaphoid, without any dislocation or comminution visible on the CT scan (long axial 1-mm cuts, high resolution)

**Exclusion criteria:**
• Patients with acute fractures of both hands
• Patients with one hand missing
• All injuries other than isolated scaphoid fractures
• Patients with radiological signs of carpal instability
• Patients with signs of any rheumatoid, osteoarthritis or polyarthritis
• Patients with previous skeletal or severe soft tissue trauma to the same wrist
• History of drug or alcohol abuse
• General or local conditions adversely affecting bone physiology
• Patients unlikely to cooperate or attend all scheduled visits
• Patients who have participated in any other device or drug clinical trial within the previous month
• Patients with physical or mental incapacity, which makes it impossible to obtain informed consent
• Patients with legal incompetence

**Table 2:** Participating clinics

• Klinik für Handchirurgie; Bad Neustadt, Germany
• Hand-, Replantations- und Mikrochirurgie; Unfallkrankenhaus Berlin-Marzahn, Germany
• HELIOS Klinikum, Unfall-, Hand- und Wiederherstellungschirurgie; Erfurt, Germany
• Hand- und Mikrochirurgie, Klinik für Unfallchirurgie; Justus-Liebig-Universität Giessen, Germany
• Universitätsklinik für Unfallchirurgie; Graz, Austria
• Klinik für Unfall- und Wiederherstellungschirurgie, Universitätsklinikum Eppendorf; Hamburg, Germany
• Universitätsklinik für Unfallchirurgie; Innsbruck, Austria
• Plastische und Handchirurgie, Universität Heidelberg, BG Unfallklinik; Ludwigshafen, Germany
• Universitätsklinik Marburg, Klinik für Unfall-, Wiederherstellungs- und Handchirurgie; Marburg, Germany
• Handchirurgie, TU München, Klinikum Rechts der Isar; München, Germany
• Unfall- und Wiederherstellungs-Chirurgie; Rosenheim, Germany
• Malteser Krankenhaus St. Josef, Klinik für Unfall- und Handchirurgie; Hamm, Germany

**Table 3:** Sample size calculation for the primary outcome (2). The table contains specified accrual and dropout rates for 6 periods. This estimate of power was based on 1000 simulations of the study with a starting value for the random number generator of 678.

End of period, time t	0	3	6	9	12	15	24
Accrual (% of total)	100	0	0	0	0	0	0
Group A exponential hazard rate	0	0.0744	0.1567	0.3054	0.0959	0.3662	0.1018
Group B exponential hazard rate	0	0.0171	0.018	0.1959	0.0744	0.231	0.154
Group A **SCREW **expected % surviving time t	100	80	50	20	15	5	2
Group B **CAST **expected % surviving time t	100	95	90	50	40	20	5
Common exponential dropout rate	0	0.0034	0.0085	0.0117	0.0107	0.0108	0.0012
							
Time t		3	6	9	12	15	24
Follow up rate (similar in both groups)		0.99	0.95	0.90	0.88	0.85	0.75
Median survival		206.903	81.08	59.209	65.067	63.975	57.826
Exponential parameter		0.0034	0.0085	0.0117	0.0107	0.0108	0.012
							
Test significance level	**0.05**						
Power (%)	**92**						
**Number needed Group A:**	**86**						
**Number needed Group B:**	**30**						

**Table 4:** Confounding factors and variables on the outcomes

• Poverty status defined by those receiving "Low income benefit" (yes/no)
• Age
• Last level of education completed
• Smoking status
• Gender
• Dominant Hand
• Patients receiving additional compensation for their injury
• Occupation
• Length of pre-injury employment
