# Supplementary material for: A prospective multi-center cohort study of acute non-displaced fractures of the scaphoid: operative versus non-operative treatment [NCT00205985]
Source: BMC Musculoskelet Disord. 2006 May 11;7:41. doi: 10.1186/1471-2474-7-41 (PMC1475583; doi:10.1186/1471-2474-7-41)
Supplement: Additional File 1 — PLDL-wrist German version [file 1471-2474-7-41-S1.PDF]

INTERVIEW (1)

Die folgenden Fragen (Abschnitt 1,2 und 3) sind als Befragung vom Untersucher auszufüllen! (Bitte nicht dem Patienten vorlegen!)
Den weiß unterlegten, einleitenden Text bitte vorlesen.

1. PLDL-wrist

PRIMARY OUTCOME

Die folgenden Fragen betreffen die Anforderungen und Belastungen in Ihrem Alltag. D. h. die berufliche Arbeit genauso wie Freizeit, Haushalt usw.
Bitte beziehen Sie alle Fragen auf die letzten 7 Tage und antworten Sie jeweils mit ‚JA' oder ‚NEIN'.
(← Bei Unklarheiten: Es interessiert nicht, was die/der Patientin/Patient kann oder eventuell könnte, sondern was sie/er wirklich getan hat.)

1.1 Haben Sie oft Lasten von mehr als 5 kg **gehoben**? ☐ **nein** ☐ **ja**

1.2 Haben Sie oft schwere Lasten von mehr als 5 kg **gezogen oder geschoben**? ☐ **nein** ☐ **ja**

1.3 Haben Sie oft schwere Lasten von mehr als 5 kg **getragen**? ☐ **nein** ☐ **ja**

1.4 Haben Sie oft in **ungünstiger Haltung** Lasten gehoben? ☐ **nein** ☐ **ja**

1.5 Haben Sie oft **vom Körper entfernt** Lasten gehoben? ☐ **nein** ☐ **ja**

1.6 Haben Sie oft Lasten von **mehr als 20 kg** gehoben? ☐ **nein** ☐ **ja**

1.7 Haben Sie oft mit den Händen und Armen **nach etwas gegriffen**? ☐ **nein** ☐ **ja**

1.8 Haben Sie oft **anhaltende drehende Bewegungen** mit Ihren Händen oder Armen gemacht? ☐ **nein** ☐ **ja**

1.9 Haben Sie oft mit den Händen oder Armen **Kraft aufgewendet**? ☐ **nein** ☐ **ja**

1.10 Haben Sie oft **kurze schnelle Bewegungen** mit den Händen oder Fingern gemacht? (z.B. Tastatur bedienen oder Schreibmaschine schreiben) ☐ **nein** ☐ **ja**

1.11 Haben Sie oft Ihr Handgelenk **gebeugt oder es länger gebeugt gelassen**? (z.B. Gitarre spielen oder Motorrad fahren) ☐ **nein** ☐ **ja**

1.12 Haben Sie oft Ihr Handgelenk **gedreht oder es länger gedreht gelassen**? (z.B. Schrauben auf-/zudrehen) ☐ **nein** ☐ **ja**

1.13 Haben Sie oft die **immer gleichen Bewegungen** mit Ihren Handgelenken gemacht? ☐ **nein** ☐ **ja**

1.14 Haben Sie oft mit den Händen **fest zugegriffen**? ☐ **nein** ☐ **ja**

1.15 Haben Sie oft sehr **kleine Gegenstände** mit Ihren Händen **gehalten oder bewegt**? (z.B. kleine Teile zusammenstecken, etwas einfädeln etc.) ☐ **nein** ☐ **ja**

1.16 Haben Sie oft eine **Computermaus** bedient? ☐ **nein** ☐ **ja**

1.17 Haben Sie sich oft mit den Händen **aufgestützt**? (z.B. beim Fahrrad fahren) ☐ **nein** ☐ **ja**

INTERVIEW (2)

Die folgenden Fragen (Abschnitt 2 und 3) sind als Befragung vom Untersucher auszufüllen! (Bitte nicht dem Patienten vorlegen!)
Den weiß unterlegten, einleitenden Text bitte vorlesen.

2. QAL — PRIMARY OUTCOME

2.1 Leistungsfähigkeit:

Jetzt eine Frage zur Einschätzung Ihrer **jetzigen Leistungsfähigkeit**: Wenn Sie Ihre beste, je erreichte Leistungsfähigkeit mit 10 Punkten bewerten: Wie viele Punkte würden Sie dann für Ihre Leistungsfähigkeit in den letzten 7 Tagen geben? **0 Punkte** bedeutet, dass Sie derzeit völlig leistungs**un**fähig sind, **10 Punkte** steht für die beste Leistungsfähigkeit.

	0	1	2	3	4	5	6	7	8	9	10	
völlig leistungs**un**fähig	☐	☐	☐	☐	☐	☐	☐	☐	☐	☐	☐	derzeit die beste Leistungsfähigkeit

2.2 Behandlungszufriedenheit:

Wie zufrieden sind Sie mit Ihrer Behandlung auf einer Schulnotenskala von ‚**Ungenügend**' bis ‚**Sehr Gut**'?

Ungenügend	Mangelhaft	Ausreichend	Befriedigend	Gut	Sehr gut
☐	☐	☐	☐	☐	☐

3. QAS - Aktivitätsstatus — PRIMARY OUTCOME

In wie weit konnten Sie in den letzten 7 Tagen folgenden Tätigkeiten nachgehen?

**3.1** Erwerbstätigkeit	im vollem Umfang ☐	teilweise ☐	gar nicht ☐	nicht erwerbstätig ☐
**3.2** Aufgaben im Haushalt	im vollem Umfang ☐	teilweise ☐	gar nicht ☐	
**3.3** Freizeitaktivitäten	im vollem Umfang ☐	teilweise ☐	gar nicht ☐	
