# Supplementary material for: A prospective multi-center cohort study of acute non-displaced fractures of the scaphoid: operative versus non-operative treatment [NCT00205985]
Source: BMC Musculoskelet Disord. 2006 May 11;7:41. doi: 10.1186/1471-2474-7-41 (PMC1475583; doi:10.1186/1471-2474-7-41)
Supplement: Additional File 2 — PLDL-wrist English translation [file 1471-2474-7-41-S2.PDF]

INTERVIEW (1)

The following question (sections 1,2 and 3) are to be filled out by the investigator during the interview! (Please do not give this form to the patient!)
Please read the introductory text (with white background) aloud to the patient.

1. PLDL-wrist

PRIMARY OUTCOME

The following questions refer to the demands and loads that you have to cope with in your daily life, that is, in your work at home and outside the home and in your leisure time.
Please relate all questions to **the last 7 days** and give a straightforward 'YES' or 'NO' answer to each question.
(← If the patient is not sure: It is not of interest what the patient can or possibly could do, but only what s/he actually did..)

1.1 Did you **lift** loads of more than 5 kg often?	☐ no	☐ yes
1.2 Did you often **push or pull** heavy loads of more than 5 kg?	☐ no	☐ yes
1.3 Did you often **carry** heavy loads of more than 5 kg?	☐ no	☐ yes
1.4 Did you often lift loads in an **awkward position**?	☐ no	☐ yes
1.5 Did you often lift loads by **holding them away from your body**?	☐ no	☐ yes
1.6 Did you often lift loads of **more than 20 kg**?	☐ no	☐ yes
1.7 Did you often try **to grab something** with your hands and arms?	☐ no	☐ yes
1.8 Did you make frequent **twisting movements over a long period** wtih your hands and arms?	☐ no	☐ yes
1.9 Did you often **apply force** with your hands or arms?	☐ no	☐ yes
1.10 Did you make frequent **short, rapid movements** with your hands or fingers? (e.g. operating a keyboard or typewriting)	☐ no	☐ yes
1.11 Did you often **bend your wrists or keep your wrists bent** for a long time? (e.g. guitar playing or motorbike riding)	☐ no	☐ yes
1.12 Did you often **twist at the wrist or keep the wrist twisted** for a long time? (e.g. screwing screws in or out)	☐ no	☐ yes
1.13 Did you **repeatedly make the same movements** with your wrists?	☐ no	☐ yes
1.14 Did you frequently have to **hold on tightly** with your hands?	☐ no	☐ yes
1.15 Did you frequently **hold or manipulate small objects** in your hands? (e.g. fitting small pieces together, threading something, etc.)	☐ no	☐ yes
1.16 Were you **operating a computer mouse** repeatedly?	☐ no	☐ yes
1.17 Did you frequently **support yourself** on your hands? (e.g. when cycling)	☐ no	☐ yes

INTERVIEW (2)

The following questions (sections 2 and 3) are to be filled out by the investigator during the interview! (Please do not give this form to the patient!)
Please read the introductory text (with white background) aloud to the patient.

2. QAL — PRIMARY OUTCOME

2.1 Physical condition:

Now a question to assess your **physical condition**. If 10 points mean that you're in top form, how many points would you have given yourself in **the last 7 days**? **0 Points** means that you were completely **incapacitated**, **10 Points** means you were in top form..

	0	1	2	3	4	5	6	7	8	9	10	
Completely **in**capacitated	☐	☐	☐	☐	☐	☐	☐	☐	☐	☐	☐	**Top form** at the time

2.2 Treatment satisfaction:

How satisfied are you with your treatment on a school grade scale of "**satisfactory**" to "**very good**"?

	poor	adequate	satisfactory	good	Very good
☐	☐	☐	☐	☐	☐

3. QAS - Activity Status — PRIMARY OUTCOME

To what extent did you pursue the following activities in the last 7 days?

**3.1** Paid employment	fully ☐	part-time ☐	not at all ☐	not in paid employment ☐
**3.2** Household duties	fully ☐	part-time ☐	not at all ☐	
**3.3** Leisure activities	fully ☐	part-time ☐	not at all ☐	
